# Long-read Sequencing and de novo Genome Assembly of Three *Aspergillus fumigatus* Genomes

**DOI:** 10.1007/s11046-023-00740-2

**Published:** 2023-05-25

**Authors:** Samuel J. Hemmings, Johanna L. Rhodes, Matthew C. Fisher

**Affiliations:** 1 Department of Infectious Disease Epidemiology, Imperial College London, London, UK; 2 Department of Medical Microbiology, Radboud University Medical Centre, Nijmegen, Netherlands

**Keywords:** *Aspergillus fumigatus*, Oxford nanopore sequencing, de novo genome assembly, Fungal genome, Azole resistance

## Abstract

*Aspergillus fumigatus* is a genetically diverse fungal species, which is near ubiquitous in its global distribution and is the major cause of the life-threatening disease invasive aspergillosis. We present 3 de novo genome assemblies that were selected to be representative of the genetic diversity of clinical and environmental *A. fumigatus*. Sequencing using long-read Oxford Nanopore and subsequent assembly of the genomes yielded 10–23 contigs with an N50 of 4.05 Mbp to 4.93 Mbp.

## Introduction

*Aspergillus fumigatus* is a globally ubiquitous environmental mould that was recently highlighted in the World Health Organization (WHO) fungal priority pathogens list as a species of critical concern [1]. *A. fumigatus* can cause invasive and chronic forms of the disease aspergillosis which results in more than 300,000 deaths per year [2]. Unfortunately, resistance of *A. fumigatus* to triazole antifungals (the first-line therapy for aspergillosis) is emerging worldwide [3].

**Fig. 1:**
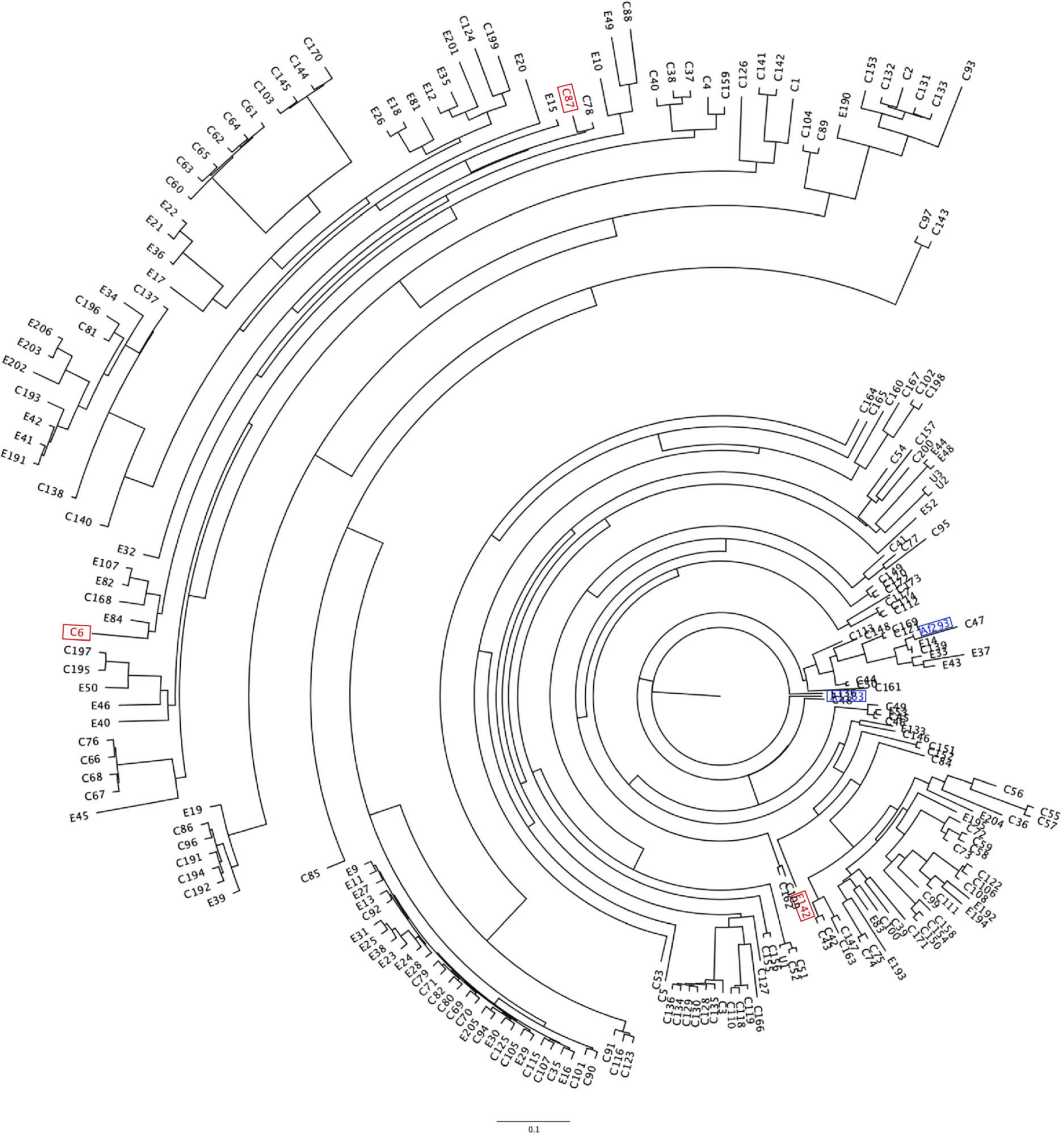
Unrooted maximum likelihood phylogenetic tree (constructed in RAxML using genome-wide SNPs) of *Aspergillus fumigatus* isolates in the UK and Republic of Ireland. The reference genomes Af293 and A1163 are highlighted in blue and C6, C87 and E142 are highlighted in red. Branch lengths refer to the mean number of substitutions per site. Average nucleotide identity (ANI) between Af293 and A1163 was calculating to be 99.69% while ANI for the two Clade A isolates (C6, C87) compared to the Clade B isolate (E142) were calculated to be 99.64% and 99.66% respectively

Previous phylogenomic analysis has shown that the population of *A. fumigatus* is genetically diverse and clusters into two clades (A and B) [4, 5]. This extensive genetic diversity provides ample opportunity for new drug resistance polymorphisms to arise. However, the current reference genomes, Af293 [6] and A1163 [7] do not span the existing known diversity. To assist in investigating why most environmental triazole resistance occurs in clade A, we have resequenced three isolates from our laboratories in-house *A. fumigatus* collection that are representative of the main diversity of *A. fumigatus* [4] (Fig. 1). Sequencing was achieved using deep nanopore sequencing to generate de novo assemblies of two clade A isolates (one of which contains the predominant resistance allele TR_34_/L98H) and a single clade B isolate. Although at time of original submission, there are currently 321 *A. fumigatus* isolates available on NCBI [8] the 3 de novo assemblies we present here are assembled into fewer contigs than > 99%. Moreover, these genomes were sequenced cheaply and inhouse using long-read sequencing and we provide a freely available, downloadable bioinformatic pipeline for research groups who also wish to produce de novo genome assemblies of fungal species with small genomes from long-read sequencing data (https://github.com/SJHemmings/afasont).

## Method

The isolates selected for sequencing were C6 (a clinical wildtype isolate from clade A, U.K.), C87 (a clinical isolate from clade A with resistant TR_34_/L98H allele, U.K.) and E142 (an environmental wildtype isolate from clade B, U.S.).

*A. fumigatus* isolates were inoculated in vented 25 cm^3^ tissue culture flasks with Sabouraud Dextrose agar (Oxoid, Hampshire, U.K.) and incubated for 48 h at 37 °C. Spores were harvested in PBS + 0.01% Tween-20 by filtration through glass wool (Thermo Fisher Scientific, Massachusetts, U.S.). Spores were centrifuged (5000 rpm for 10 min) and resuspended in Yeast Cell Lysis Solution (Biosearch Technologies, Hoddesdon, U.K.) and vortexed at maximum speed for 10 min with 1.0 mm zirconia/silica beads (Thistle Scientific, Glasgow, U.K.). The suspension was then centrifuged (14,000 rpm for 2 min) and supernatant was removed and treated with RNase Cocktail™ Enzyme Mix (Thermo Fisher Scientific) according to the manufacturer’s instruction. DNA was then isolated on spin columns using AW1 and AW2 wash buffers (Qiagen, Venlo, Netherlands) and eluted in nuclease free water. To achieve the required relative absorption ratios for Oxford Nanopore sequencing and to concentrate DNA, additional washing steps were carried out using 0.6X AMPure Reagent (Beckman Coulter, California, U.S.) and 70% ethanol. An SRE XS kit (Pacific Biosciences, California, U.S.) was used to deplete any remaining reads below 10 kb. For quality control, DNA was visualised using Genomic DNA Screentape on a TapeStation (Agilent Technologies, California, U.S.) to ensure the average DNA length was above 20 kbp. 1 μg of DNA was prepared for sequencing using an SQK-LSK110 ligation sequencing kit (Oxford Nanopore Technologies, Oxford, U.K.) and NEBNext Companion Module (New England Biolabs, Massachusetts, U.S.) following the manufacturer’s instructions. Isolates were sequenced on a minION using an R10.4 flow cell (Oxford Nanopore Technologies) for a total of 18 h.

Live base calling was performed using Guppy v6.3.9. Porechop v0.2.4 [9] and NanoLyse v1.2.1 [10] were used to remove adapters and sequences and CS DNA (Oxford Nanopore Technologies) from raw fastq files. Reads were filtered using NanoFilt v2.8.0 [10] to remove reads with a quality score below Q10 or less than 1 kbp in length. Reads passing quality control were used for de novo assembly using Canu v2.2 [11] with a specified genome length of 29 Mbp. The assembled genomes were polished with Pilon v1.24 [12] using Illumina paired-end reads (150 bp) sequenced on a NovaSeq 6000 SP v1.5 (185X coverage for C6, 29X coverage for C87 and 51X coverage for E142) at the Earlham Institute (UK). Illumina paired-end reads can be accessed from the European Nucleotide Archive at EMBL-EBI under accession code PRJEB27135. The number of tRNA and protein coding genes within the assembles were then estimated using tRNAscan-SE v2.0.9 [13] and AUGUSTUS v3.8.0 [14]. Genome completeness was then predicted using BUSCO coupled with the ascomycota_odb10 lineage dataset [15]. The full pipeline (‘afasont’) used to generate these assemblies is available from https://github.com/SJHemmings/afasont. Finally, BLAST v2.12.0 + [16] analysis was used to screen the individual contigs for contamination.

## Genome Details

After passing through quality control, raw reads from the minION showed coverage of 82X for C6, 73X for C87 and 149X for E142. Isolates C6, C87 and E142 were then assembled into genomes of 29,266,253 bp, 28,591,451 bp and 28,644,426 bp in length.

C6 was assembled into 10 contigs with an N50 of 3.99 Mbp with a longest contig of 4.93 Mbp. AUGUSTUS [14] predicted 8,802 protein coding genes and tRNAscan-SE [13] detected 211 genes which encode for transfer RNA. Using the ascomycota_odb10 lineage dataset, BUSCO estimated genome completeness to be 98.5% [15].

C87 assembled into 23 contigs with an N50 of 2.55 Mbp, the longest contig reached 4.05 Mbp in length. AUGUSTUS [14] found 8830 protein coding genes and tRNAscan-SE [13] estimated there were 200 genes which encode for tRNA. Genome completeness was predicted to be 97.6% using BUSCO [15].

E142 was assembled into 15 contigs and has a N50 of 2.67 Mbp, the longest contig was 4.20 Mbp. 8,806 protein coding genes were found by AUGUSTUS [14] and tRNAscan-SE [13] detected 208 genes which encode for tRNA. 98.1% genome completeness was estimated using BUSCO [15].

Raw read files sequenced with Oxford Nanopore Technologies and de novo genome assemblies can be accessed from the European Nucleotide Archive at EMBL-EBI under the accession code PRJEB59410. The sequence accessions for the individual assemblies are: CASBLW01 (GCA_949125545) (C6); CASBLU01 (GCA_949125165) (E142); CASBLV01 (GCA_949125185) (C87).
